# Analysis and prediction of cancerlectins using evolutionary and domain information

**DOI:** 10.1186/1756-0500-4-237

**Published:** 2011-07-20

**Authors:** Ravi Kumar, Bharat Panwar, Jagat S Chauhan, Gajendra PS Raghava

**Affiliations:** 1 Bioinformatics Centre Institute of Microbial Technology, Sector-39A, Chandigarh, India

## Abstract

**Background:**

Predicting the function of a protein is one of the major challenges in the post-genomic era where a large number of protein sequences of unknown function are accumulating rapidly. Lectins are the proteins that specifically recognize and bind to carbohydrate moieties present on either proteins or lipids. Cancerlectins are those lectins that play various important roles in tumor cell differentiation and metastasis. Although the two types of proteins are linked, still there is no computational method available that can distinguish cancerlectins from the large pool of non-cancerlectins. Hence, it is imperative to develop a method that can distinguish between cancer and non-cancerlectins.

**Results:**

All the models developed in this study are based on a non-redundant dataset containing 178 cancerlectins and 226 non-cancerlectins in which no two sequences have more than 50% sequence similarity. We have applied the similarity search based technique, i.e. BLAST, and achieved a maximum accuracy of 43.25%. The amino acids compositional analysis have shown that certain residues (e.g. Leucine, Proline) were preferred in cancerlectins whereas some other (e.g. Asparatic acid, Asparagine) were preferred in non-cancerlectins. It has been found that the PROSITE domain "Crystalline beta gamma" was abundant in cancerlectins whereas domains like "SUEL-type lectin domain" were found mainly in non-cancerlectins. An SVM-based model has been developed to differentiate between the cancer and non-cancerlectins which achieved a maximum Matthew's correlation coefficient (MCC) value of 0.32 with an accuracy of 64.84%, using amino acid compositions. We have developed a model based on dipeptide compositions which achieved an MCC value of 0.30 with an accuracy of 64.84%. Thereafter, we have developed models based on split compositions (2 and 4 parts) and achieved an MCC value of 0.31, 0.32 with accuracies of 65.10% and 66.09%, respectively. An SVM model based on Position Specific Scoring Matrix (PSSM), generated by PSI-BLAST, was developed and achieved an MCC value of 0.36 with an accuracy of 68.34%. Finally, we have integrated the PROSITE domain information with PSSM and developed an SVM model that has achieved an MCC value of 0.38 with 69.09% accuracy.

**Conclusion:**

BLAST has been found inefficient to distinguish between cancer and non-cancerlectins. We analyzed the protein sequences of cancer and non-cancerlectins and identified interesting patterns. We have been able to identify PROSITE domains that are preferred in cancer and non-cancerlectins and thus provided interesting insights into the two types of proteins. The method developed in this study will be useful for researchers studying cancerlectins, lectins and cancer biology. The web-server based on the above study, is available at http://www.imtech.res.in/raghava/cancer_pred/

## Background

Basically 'Lectins' derived from the Latin word "*legere*" which means "to select", are the biomolecules that specifically recognize and bind to carbohydrates moieties present on other proteins e.g. glycoproteins or lipids e.g. glycolipids [1]. Lectins have been known to be involved in numerous biological events e.g. host-pathogen interactions, cell-cell recognition, complement activation pathways, cell cycle regulation, apoptosis etc. Most lectins are highly specific and selective in recognizing the sugar moieties present on other proteins and bind to them reversibly and non-covalently without inducing any change in the bound carbohydrates [2]. These glycoproteins are generally classified into five groups based on the monosaccharides for which they exhibit the highest affinity. These monosaccharides are mannose, galactose/*N*-acetylgalactosamine, *N*-acetylglucosamine, fucose, and sialic acid [3]. Not only do lectins vary significantly in their individual functional roles, but they are also diverse in their sequences, structures, binding site architectures, quaternary structures, carbohydrate affinities and specificities as well as in their potential applications [4].

Cancerlectins are known to play various important roles in cancer metastasis [5-7]. Several lines of evidence implicate tumour cell lectins in cellular interactions such as adhesion, cell growth, tumour cell differentiation, metastasis and cellular infection [8,9]. The carbohydrate-binding properties of lectins have been used to identify tumour specific patterns in cancer cells, e.g. Helix Pomatia agglutinin binding is a useful prognostic indicator in colorectal carcinoma [10-13]. Many lectins act as therapeutic lectins preferentially binding to cancer cell membranes or their receptors causing cytotoxicity, apoptosis, and inhibition of tumour growth [14]. Galectin is known to play a role in infections as well as act as modulator of tumour formation [9,15]. Galectin-3 is also known to enhance the metastasis potential in human breast carcinoma BT549 and in cancer apoptosis [16,17]. Mistletoe lectins are known to induce apoptosis and telomerase inhibition in Human A253 cancer cells [18].

Cancerlectins are known in terms of their source, class, domain, fold class, quaternary structure and carbohydrate specificity but the method to distinguish cancerlectins from lectins or non-cancerlectin is still missing [19]. Results of similarity based techniques like BLAST and FASTA are reliable only when the query sequence has high sequence similarity with experimentally annotated proteins [20-22]. In this study we systematically analyzed cancerlectins and non-cancerlectins and developed a method for their classification. We developed a Support Vector Machine (SVM) based prediction method, CancerPred for annotating cancerlectins on the basis of amino acid composition and evolutionary information using PSSMs, also having information about the specific PROSITE domains found in the two types of proteins.

## Methods

### Datasets preparation

We downloaded 509 cancerlectin protein sequences from cancerlectinDB database (http://proline.physics.iisc.ernet.in/cgi-bin/cancerdb/input.cgi). After removing the proteins having 100% sequence similarity using the CD-HIT program, we obtained 385 sequences which formed the positive dataset. For a negative dataset, we searched the UniProt database (http://www.uniprot.org/) with the keyword "lectin" and a total number of 1550 non-redundant sequences were obtained. These were further filtered by excluding the sequences containing the keywords "similar", "fragment", "putative" and "probable", resulting in 891 lectin sequences. Seventy-one sequences were found to be common to cancerlectins and lectins. These sequences were then removed from lectins, reducing the number of lectins to 820. To balance the datasets, a total of 385 sequences were randomly selected from the 820 lectin sequences, to equalize the number of lectins to the initial number of cancerlectins. Furthermore, to make non-redundant datasets, the CD-HIT program was used at 50% cutoff resulting in 178 cancerlectin and 226 non-cancerlectin sequences.

### Subset sequences similarity

Although we had removed the 100% identical sequences (71 from non-cancerlectins) and reduced the redundancy up to 50% by using CD-HIT program, there were chances of similarity between the two datasets (subsets). To determine the similarity between cancer and non-cancerlectins, we employed the BLAST tool using the non-cancerlectins as test sequences against a database of cancerlectins with an E value cut-off of 0.001. Out of 226 queries, a total of 145 hits were found, which confirmed that there was 64.15% sequence similarity between non-cancerlectin and cancerlectin datasets.

### Five-fold cross validation

Evaluation of newly-developed methods is a big challenge in Bioinformatics. One of the most common techniques for model evaluation is the Jack-knife test or leave-one out cross-validation (LOOCV) [23-25]. In this technique, one sequence is used for testing and the remaining ones are used for training and the entire process is repeated in such a way that each sequence is used once for testing. Although it is one of the best techniques, it is very time consuming and computationally demanding. Therefore, we used the five-fold cross validation technique where the whole set of sequences is randomly divided into five sets. One set was used for testing and the remaining sets were used for training. This process was repeated five times in such a way that each test set was used once for testing [26,27]. The final performance was the average of the performances of the five sets.

### Evaluation parameters

A set of parameters used to evaluate the performance of the various methods is briefly described below.

#### 1. Sensitivity

Sensitivity, or percentage coverage, is the percentage of cancerlectins correctly predicted as "cancerlectins".

#### 2. Specificity

Percentage of non-cancerlectins correctly predicted as "non-cancerlectins".

#### 3. Accuracy

Percentage of overall correctly predicted proteins (cancer and non-cancerlectins).

#### 4. Matthew's correlation coefficient (MCC)

It is the statistical parameter used to assess the quality of predictions and to correct the imbalance in the data. It is calculated as follows:

Where TP is the number of correctly predicted proteins in the positive dataset (cancerlectins) and TN is the number of correctly predicted proteins in the negative dataset (non-cancerlectins), whereas FP is the number of wrongly predicted proteins in the positive dataset and FN is the number of wrongly predicted proteins in the negative dataset. For the evaluation of a new prediction method MCC is considered the most robust parameter [28]. An MCC value of '1' corresponds to a prefect prediction and '0' corresponds to a completely random prediction. The limitation of the above-described parameters is that they are threshold dependent and require proper optimization for good performance. We optimized these parameters manually and selected the ones that gave the best performance. A known threshold independent parameter is the Receiver Operating Curve (ROC), which is a plot of the true positive rate (TP/TP+FN) *versus *the false positive rate (FP/FP+TN). The area under the curve (AUC) gives a single value to evaluate the performance of a method. We used the SigmaPlot 11.0 package for plotting the ROC and calculating the AUC.

### Support vector machine (SVM)

In this study we employed a highly successful machine learning technique known as "Support Vector Machine", which is freely available at http://www.cs.cornell.edu/People/tj/svm_light/. SVM is based on the structural risk minimization principle of statistics learning theory [29]. SVM is a set of related supervised learning methods used for classification and regression. It allowed us to choose a number of parameters and kernels (e.g. Linear, Polynomial, Radial and sigmoid) or any other user-defined kernel. We implemented the SVM^light ^version 6.01 package of SVM and learning was carried out by using three (linear, polynomial and radial basis function) kernels [30]. SVM takes a set of free vectors as input, along with their output, which is used for training the models. The trained model was used for the prediction of non-annotated proteins [31]. In this work, we selected the learning option -z (c) for classification purposes. The SVM training was performed by optimizing various kernel function parameters and the value of the regularization parameter C. Preliminary tests showed that the radial basis function (RBF) kernel provided better results than other kernels. Therefore, the RBF kernel was used for all the experiments. In the RBF kernel, we first optimized parameters for gamma -g (0.0005 to 25), then further optimized -c (-0.1 to 10) and finally the cost factor -j (1 to 10). In this study, we have used amino acid composition, dipeptide composition, split compositions (2 and 4), PSSM and PSSM-PROSITE domains as input vectors in the SVM-based machine learning technique.

### Protein features

The aim of calculating the composition of proteins is to convert the variable length of protein sequences into fixed length feature vectors. This is a crucial step because the SVM machine learning technique requires fixed length patterns.

#### Amino acid composition

Amino acid composition is the fraction of each of amino acid in a protein sequence and provides vector of 20 dimensions. The SVM was provided with these 20 dimension vectors encapsulating the amino acid composition of proteins.

#### Dipeptide composition

Dipeptide composition was used to give global information about each protein sequence and it gives a fixed length pattern of 400 (20 **× **20) features, one for each dipeptide. The dipeptide composition incorporates the fraction of amino acids as well as their local order i.e order of amino acids in a protein sequence. In this way, dipeptide composition is more informative than amino acid composition.

#### Split amino acid composition

Split composition was used to detect conserved residues or signal peptides in any terminal of the given protein sequences [32-34]. In case of split amino acid composition, a sequence was divided into non-overlapping fragments and amino acid composition of each fragment was calculated independently. Thus, the dimension of the final input vector was N **× **20, where N is the number of fragments. In this study, proteins sequences were divided into (i) two parts (N = 2) and (ii) four parts (N = 4) generating 40 and 80 input dimensions, respectively.

#### Evolutionary information in the form of PSSM profiles

In this study, PSSM profiles were generated using PSI-BLAST [29] to search a protein sequence against the Swiss-Prot database with an E-value cutoff of 0.001. A profile contains the probability of occurrence of each amino acid and of insertion/deletion at every position along the sequence. In this way, a PSSM was considered as a measure of residue conservation at a given location. This meant that evolutionary information for each amino acid was encapsulated in a vector of 20 dimensions, where the size of the PSSM for a protein with M residues is 20 **× **M, where M is the length of the target sequence and each element represents the frequency of occurrence of each of the 20 amino acids [35].

Next, each element of the matrix (20 **× **M) was scaled to the range of 0-1 using a sigmoid function. Further, in order to obtain an input of fixed length, these normalized PSSMs (20 **× **M) were used to generate a 400-dimensional input vector by summing all rows in the PSSM corresponding to each type of amino acid in the sequence. Finally, each element in the input vector was divided by the length of the protein sequence resulting in a matrix of 400 (20 **× **20) elements, which was used as input vector for training the SVM.

#### PROSITE domains in cancer and non-cancerlectins

PROSITE is a database of families and domains found in various proteins. During evolution, it is apparent that all protein families conserve some portions of protein sequences for efficient function and/or stability of three-dimensional structure, which distinguish family members from other proteins. InterProScan (IPRScan) is a Perl based stand-alone tool that combines different protein signature-recognition methods into a single platform [36]. PROSITE database is an integral part of InterProScan. In this work, we searched and analyzed PROSITE domains in cancer and non-cancerlectins using ProfileScan method of InterProScan tool (version 4.4.1). Out of 178 cancer and 226 non-cancerlectins, only 99 and 122 sequences were found to contain one or more PROSITE domains, respectively. A total of 151 and 200 PROSITE domains were found in cancer and non-cancerlectin dataset, respectively.

## Results and discussion

### Analysis of amino acid composition

**Figure 1:**
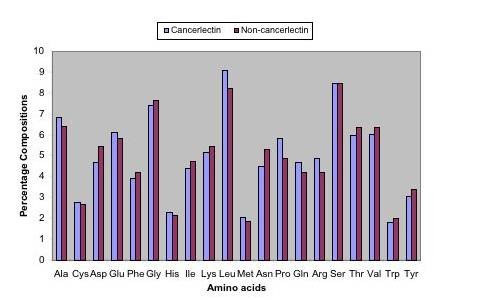
**Amino acid compositions of cancer and non-cancerlectins**. Comparative frequencies of 20 amino acids in cancerlectins and non-cancerlectins.

We analyzed the amino acid composition of both cancer and non-cancerlectins proteins with the help of the Copid (http://www.imtech.res.in/raghava/copid/) web server. As shown in Figure 1, the frequency of Ala, Glu, Leu, Pro, Gln and Arg is higher in cancerlectins, while the frequency of Asp, Phe, Ile, Lys, Asn, Thr, Val and Tyr is higher in non-cancerlectins. There are major differences in composition of proline between cancerlectins (high) and non-cancerlectins. This means that cancer and non-cancerlectins can be distinguished on the basis of their amino acid compositions. We also analyzed the statistical significance of the differences observed in the amino acid composition, in terms of p-value. We have noticed that Aspartic acid, Lysine, Leucine, Asparagine, Proline and Arginine vary significantly in their composition in cancer and non-cancerlectins, with p-values of 0.002, 0.007, 0.009, 0.003, 0.007 and 0.006 respectively [Additional file 1 Supplementary Table S1].

### Sequence similarity using BLAST

The most commonly used method for predicting the function of a new protein is BLAST. It is a sequence similarity based method and identifies regions/segments in the query protein which are similar to the target sequences. Thus, we have applied a BLAST-based approach for discriminating between cancer and non-cancerlectins at E-values ranging from 10^-1 ^to 10^-5^. In this study, we used BLAST for predicting cancerlectin proteins. We used a 5-fold cross-validation where four sets of cancer and non-cancerlectins were used to create a database whereas cancerlectins of the corresponding fifth test set were searched against this database. This process was repeated five times so the BLAST search was performed once for each cancerlectin sequence. We calculated the performance of BLAST in terms of percentage coverage, which indicated the number of correct predictions in a test set. As shown in Table 1, we achieved a maximum accuracy of 43.50% at an E-value cutoff of 0.1. It is clear that BLAST is inefficient in distinguishing between cancer and non-cancerlectins. So there is a need to develop models based on machine learning technique to discriminate cancer and non-cancerlectins with a high accuracy [Additional file 1 Supplementary Table S2].

**Table 1:** Performance of BLAST on positive dataset of 178 cancerlectins at different E-values cutoffs

E-value	Total Proteins	Total Hits	No Hits	Correct Hits	% coverage
0.1	178	150	28	77	43.25

0.01	178	144	34	74	41.57

0.001	178	143	35	74	41.57

0.0001	178	142	36	74	41.57

0.00001	178	140	38	72	40.44

0.000001	178	140	38	72	40.44

### SVM models of amino acid and dipeptide compositions

**Figure 2:**
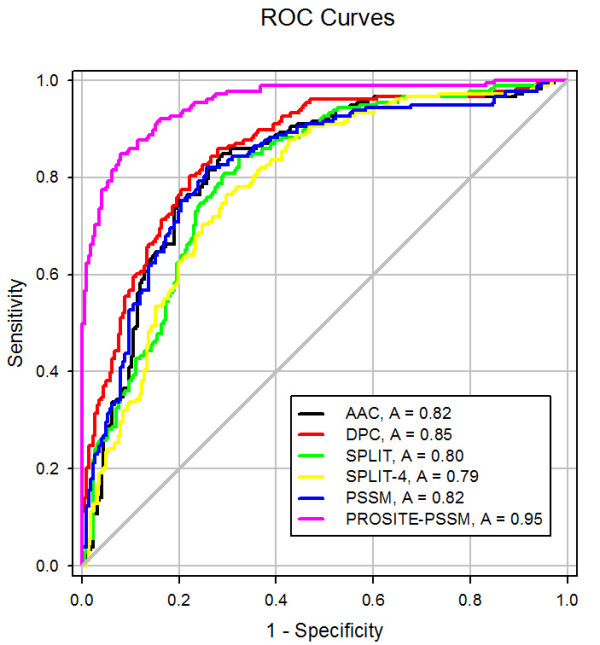
**ROC plots for various models**. Performance of different SVM modules (amino acid, dipeptide, split-2 & 4, PSSM, PSSM-PROSITE) by Receiver Operating Characteristic (ROC) plot. In the graph, "A" signifies the "AUC" value of the respective models.

It can be inferred from the above analysis that the cancer and non-cancerlectins can be distinguished on the basis of their composition. Hence, we developed a SVM-based model using amino acid composition for predicting cancer and non-cancerlectins and achieved a maximum MCC value of 0.32 using the RBF kernel. Previous studies have shown that dipeptide compositions can be successfully used for prediction of subcellular localization of human protein [37,38]. It was observed that methods based on dipeptide composition performed better than amino acid composition based methods because dipeptide also provided information about the local order of the residues in addition to the amino acid composition. Hence, in this study we developed an SVM module using dipeptide composition and achieved a maximum MCC value of 0.30 (with 64.84% accuracy) using RBF kernel [Table 2]. We have further achieved AUC values 0.82 and 0.85 for amino acid composition and dipeptide composition respectively [Figure 2]. Since the five-fold cross validation technique provides the average accuracy over five sets, the standard error of mean associated with the final accuracy have been calculated (Additional file 1 supplementary Table S3, S4).

**Table 2:** Performance of various modules of SVM developed by using Amino acid, dipeptide, split (2 and 4-part), PSSM and PSSM-PROSITE domain based input features

Methods	Threshold	Sensitivity	Specificity	Accuracy	MCC
Amino acid Composition	-0.3	67.97	64.15	65.84	0.32

Dipeptide Composition	-0.3	67.27	62.84	64.84	0.30

Split based Composition (2-part)	-0.3	66.32	64.18	65.10	0.31

Split based Composition (4-part)	-0.5	65.12	66.85	66.09	0.32

Position-Specific Scoring Matrix	-0.2	67.92	68.57	68.34	0.36

PSSM with 14 PROSITE domains	-0.1	68.00	69.90	69.09	0.38

(For details see Additional file 1 Tables S3 to S8)

### Split amino acid composition

Split amino acid compositions have been used successfully in the past to differentiate two types of proteins with peptide signals at N or C-terminal. In order to utilize the compositional biasness in the termini of cancer and non-cancerlectins, we developed SVM modules using split amino acid compositions. We used the split (2 and 4) approach by dividing the protein into two and four equal parts and calculating the amino acid compositions. This approach achieved MCC values of 0.31 and 0.32 (with accuracies 65.10% and 66.09%), respectively [Table 2] (Additional file 1 supplementary Table S5, S6). In terms of AUC, we achieved 0.80 and 0.79 values for split-2 and split-4 compositions, respectively [Figure 2].

### PSSM based SVM models

It has been shown in several studies the evolutionary information obtained using multiple sequence alignment provides more comprehensive information about a protein than a single sequence [31]. Earlier, PSSM matrices having multiple sequence alignment information were used for developing methods for alpha, beta and gamma-turn prediction in protein sequences [39-41]. In this study, PSSMs are used for predicting cancerlectins. Firstly, we created a PSSM profile for each protein using a PSI-BLAST search against the SwissProt database with three iterations and with an E-value cutoff of 0.001. Secondly, we computed a vector of 400 dimensions from the PSSM. Finally, an SVM model was developed from the PSSM. The model achieved a maximum accuracy value of 68.34% with an MCC value of 0.36 and an AUC value of 0.80 [Figure 2]. This clearly demonstrates that a PSSM provides more information than a single sequence and is useful for predicting cancerlectins [Table 2] (Additional file 1 supplementary Table S7).

### PROSITE domains

We selected the 14 most distinguishable domains present in either cancer or non-cancerlectins datasets: four (PROSITE ids: PS50049, PS50217, PS50287 and PS50915) in cancerlectins and ten (PROSITE ids: PS51127, PS50927, PS50228, PS50068, PS50092, PS50234, PS50853, PS50948, PS51115 and PS51117) in non-cancerlectins [Table 3]. The domains "PS50915" and "PS50049", which correspond to "crystalline beta-gamma" and "TNF family signature" respectively, were exclusively found in cancerlectins. Out of the ten specific domains for non-cancerlectins, only PS50228 was found once in cancerlectins.

**Table 3:** The PROSITE domain in 178 cancerlectins and 226 non-cancerlectins with their respective rate of occurrence in two classes of proteins, with descriptions of domain name

PROSITE ID of the Domain	No. of domains in cancerlectins	No. of domains in lectins	Class	Description
**PS50049**	**2**	**0**	**Cancerlectins**	**TNF Family Signature**

**PS50217**	**2**	**0**	**Cancerlectins**	**Basic Leucine Zipper domain**

**PS50287**	**2**	**0**	**cancerlectins**	**SRCR domain signature**

**PS50915**	**7**	**0**	**cancerlectins**	**Crystallin beta & gamma**

PS51127	0	3	lectins	Big-1(Bacterial Ig like-1)

PS50927	0	4	lectins	Bulb-type lectin domain

PS50228	1	7	lectins	SUEL-type lectin domain

PS50068	0	2	lectins	LDL-receptor class-A domain

PS50092	0	2	lectins	Thrombospondin Type-1

PS50234	0	2	lectins	VWFA domain

PS50853	0	2	lectins	Fibronectin type-III domain

PS50948	0	2	lectins	PAN/apple domain

PS51115	0	2	lectins	Laminin IV domain

PS51117	0	2	lectins	Laminin-N terminal domain

The bold PROSITE domains show that these domains were reported in Cancerlectins only.

### SVM model using evolutionary information and PROSITE domains

We generated a vector of 414 dimensions which contains 400 PSSM features and 14 features for the selected 14 PROSITE domains. Finally, a SVM-based classifier was developed using 414 features, 400 from PSSM profile and 14 from domains, which achieved an accuracy level of 69.09% with MCC value of 0.38 [Table 2] (Additional file 1 supplementary Table S8). As shown in Figure 2, the highest AUC value (0.95) is achieved with the PSSM-PROSITE domain SVM.

### Performance on random dataset

In this study, we built random datasets of cancer and non-cancerlectins sequences by replacing 50% of cancerlectins into non-cancerlectins and vice-versa resulting in two new datasets, each with 50% cancerlectins and non-cancerlectins. We calculated the amino acid composition and achieved an accuracy of 54.38% with an MCC value of 0.09 [Additional file 1 Supplementary Table S10]. This shows that our original SVM models were built on concrete information from amino acid, dipeptide, split compositions and were capable of discriminating cancer and non-cancerlectins with high accuracy.

### Comparison with existing methods

It is important to compare the performance of a newly developed method with that of other existing methods. In the past, a number of methods have been developed related to lectins e.g. sugar-binding site in proteins, prediction of secondary structure of legume lectins etc. but to the best of author's knowledge, there was no method that could discriminate cancerlectins from lectins/non-cancerlectins. We developed a novel method to distinguish cancerlectins from non-cancerlectins with high precision.

## Web server

We developed a webserver, CancerPred for the prediction of cancerlectins which is freely available at URL http://www.imtech.res.in/raghava/cancer_pred/. It is developed under Solaris environment on a SUN system, using CGI-PERL as programming language. This server predicts whether a protein will be a cancerlectin or a non-cancerlectin. The web server is user-friendly and many options e.g. amino acid, dipeptide, split composition-based methods etc. For multiple sequence submissions, the "submit1" option should be chosen whereas for 'PSSM' and 'PSSM-PROSITE' based predictions, the "submit2" option has to be selected, with single sequence as input.

## Discussion

Due to the rapid advancement in genomics and proteomics, a tremendous amount of data is generated every year. Functional annotation of all these proteins is not possible by using only experimental approaches, as they are laborious, costly and time-consuming. Therefore, computational methods are required to fill this gap. The functional annotation of all proteins is not possible at a time. It is therefore important to concentrate on a single class of functionally important proteins. Cancerlectins represent an important class of proteins involved in various types of cancer metastasis, differentiation etc. Therefore, it is very important to distinguish cancerlectins from lectins (non-cancerlectins), which are growing at a tremendous rate (~5280 lectin sequences annually). In the past, predictions of (I) Sugar-binding sites on proteins (II) Secondary structure of various legume lectins have been reported [42-44]. The quaternary associations in legume lectins and mutagenesis and docking studies have also been reported [45,46]; but there was no method which could distinguish cancerlectins from non-cancerlectins. We tried to predict cancerlectins using existing techniques such as BLAST, obtaining poor results, both in terms of accuracy and percentage coverage. Thus, the BLAST-based prediction method is unsuccessful in the case of cancerlectin prediction.

In this study, a systematic attempt has been made to predict cancerlectins. In amino acid composition, we collected information about the frequency of the 20 types of amino acids and used it in machine learning technique. However, this approach provides information only about the amino acid frequency, but not about the local order of amino acids. To provide information about both frequency and local order of amino acids, dipeptide composition can be used as input. To check the presence of any signal peptide present in cancerlectins, we used the split amino acid composition in the form of SVM input vectors. In our composition-based SVM models, the overall accuracy of amino acid, dipeptide, split-based compositions were comparatively similar (~65%) with MCC values of 0.32, 0.30, 0.31 respectively. The PSSM-based evolutionary information provides better information [47] hence we also made an attempt to develop a method using evolutionary information for predicting cancerlectins. The PSSM results showed that the SVM model achieved an MCC value of 0.36 with 68.34% accuracy. This demonstrates that evolutionary information is important for predicting cancerlectin proteins. PROSITE-domains along with PSSMs were used to train and develop the further SVM modules to predict cancerlectins. A total of 14 PROSITE domains (4 from cancerlectins and 10 from non-cancerlectins) were used for the model development. This SVM module achieved the highest accuracy of 69.09% with an MCC value of 0.38. Certain PROSITE domains, e.g. PS50287 and PS50217 referring to "SRCR domain" and "Basic Leucine Zipper domain" respectively were exclusively found in cancerlectins. PS50927 and PS50228 referred as "Bulb type lectin domain" and "SUEL-type lectin domain" respectively was abundant in non-cancerlectins. The Annexin (PROSITE domain - PS00223), which is only found among cancerlectins in the whole dataset, is involved in various biological processes including various cancers e.g. prostate, colorectal, breast and pancreatic cancer etc [48-51]. Crystalline beta-gamma (PS50915) is the structural protein mainly found in the lens of the vertebrate eye and it is reported to play a role in oncogenesis of the lens [52]. Improvements in prediction efficiency suggest that PROSITE domain information has an important role in protein discrimination, as cancerlectins and non-cancerlectins differ in their PROSITE domain compositions.

## Conclusion

This work attempts to predict cancerlectins, from a pool of non-cancerlectins or simple lectins. We analyzed the protein sequences of cancer and non-cancer lectins and selected the distinguishable patterns e.g. amino acid, dipeptide and split compositions. The patterns are based on evolutionary information obtained by PSSM, and PROSITE domain with PSSM. We used these patterns as input features in SVM, a machine learning technique used for classification and regression studies. We were able to model an efficient classifier from PROSITE-PSSM based approach. A web server CancerPred has been developed on the SVM modules.

## List of abbreviations

AI: Artificial Intelligence; AUC: Area Under Curve; BLAST: Basic Local Alignment Search Tool; MCC: Matthew's Correlation Coefficient; PSI-BLAST: Position Specific Interactive BLAST; PSSM: Position Specific Scoring Matrix; SVM: Support Vector Machine.

## Competing interests

The authors declare that they have no competing interests.

## Authors' contributions

RK created the dataset and developed the SVM models used for developing the web server. GPSR conceived the project, coordinated it and refined the final manuscript drafted by RK, BP and JSC. Web server was developed by RK and BP. All authors have read and approved the manuscript.

## Supplementary Material

Additional file 1 **Supplementary Tables. Table S1**: p-values for compositional differences in cancerlectins and non-cancerlectins residues. The amino acid compositions of cancer and non-cancerlectins and p-value of composition difference in between the two types of proteins. The bold values show the significant difference in composition of cancer and non-cancerlectins, in term of p-values. **Table S2**: Performance of BLAST on individual test sets of cancerlectins at E-value cutoff of 0.001. The result of BLAST search on dataset of cancerlectins. The total hits means the total number of hits found for a test set in BLAST search, no hits is the number of proteins that did not get any hit whereas correct hits shows the proteins whose top most hit belongs to the cancerlectin class. The percentage coverage indicates the proteins that were predicted as cancerlectins from the BLAST search. **Table S3: **The performance of SVM model (Learning Parameter: -z c -t 2 -g 0.01 -c 0.5 -j 1) using Amino acid composition method. This table describes the performance of amino acid composition based SVM model at each threshold (-1 to 1), providing sensitivity, specificity, accuracy and standard error and MCC. **Table S4: **The performance of SVM model (Learning Parameter: -z c -t 2 -g 0.001 -c 5 -j 1) using Dipeptide composition method. This table describes the performance of dipeptide composition based SVM model at each threshold (-1 to 1), providing sensitivity, specificity, accuracy and standard error and MCC. **Table S5: **The performance of SVM model (Learning Parameter: -z c -t 2 -g 0.001 -c 1 -j 1) using Split amino acid composition (2-part) method. This table describes the performance of split amino acid (2-part) composition based SVM model at each threshold (-1 to 1), providing sensitivity, specificity, accuracy and standard error and MCC. **Table S6: **The performance of SVM model (Learning Parameter: -z c -t 2 -g 0.0001 -c 1 -j 1) using Split amino acid composition (4-part) method. This table describes the performance of split amino acid (4-part) composition based SVM model at each threshold (-1 to 1), providing sensitivity, specificity, accuracy and standard error and MCC. **Table S7: **The performance of SVM model (Learning Parameter: -z c -t 2 -g 7 -c 1 -j 1) using PSSM-based method. This table describes the performance of PSSM based SVM model at each threshold (-1 to 1), providing sensitivity, specificity, accuracy and standard error and MCC. **Table S8: **The performance of SVM model (Learning Parameter: -z c -t 2 -g 7 -c 5 -j 1) using PSSM-PROSITE Domain based method. This table describes the performance of PSSM-PROSITE domain based SVM model at each threshold (-1 to 1), providing sensitivity, specificity, accuracy and standard error and MCC. **Table S9: **All reported PROSITE domains in cancer and non-cancerlectins. All reported domains reported in cancer and non-cancerlectins with their frequency of occurrence. A total of 151 and 200 PROSITE domains were reported in cancer and non-cancer lectins respectively. **Table S10: **The performance of amino acid composition based SVM model (Learning Parameter: -z c -t 2 -g 0.01 -c 0.5 -j 1) using random dataset of cancer and non-cancerlectins. This table describes the performance of amino acid composition based SVM model at each threshold (-1 to 1), providing sensitivity, specificity, accuracy and MCC. Click here for file
