# Supplementary material for: Analysis and prediction of cancerlectins using evolutionary and domain information
Source: BMC Res Notes. 2011 Jul 20;4:237. doi: 10.1186/1756-0500-4-237 (PMC3161874; doi:10.1186/1756-0500-4-237)
Supplement: Additional file 1 — Supplementary Tables. Table S1: p-values for compositional differences in cancerlectins and non-cancerlectins residues. The amino acid compositions of cancer and non-cancerlectins and p-value of composition difference in between the two types of proteins. The bold values show the significant difference in composition of cancer and non-cancerlectins, in term of p-values. Table S2: Performance of BLAST on individual test sets of cancerlectins at E-value cutoff of 0.001. The result of BLAST search on dataset of cancerlectins. The total hits means the total number of hits found for a test set in BLAST search, no hits is the number of proteins that did not get any hit whereas correct hits shows the proteins whose top most hit belongs to the cancerlectin class. The percentage coverage indicates the proteins that were predicted as cancerlectins from the BLAST search. Table S3: The performance of SVM model (Learning Parameter: -z c -t 2 -g 0.01 -c 0.5 -j 1) using Amino acid composition method. This table describes the performance of amino acid composition based SVM model at each threshold (-1 to 1), providing sensitivity, specificity, accuracy and standard error and MCC. Table S4: The performance of SVM model (Learning Parameter: -z c -t 2 -g 0.001 -c 5 -j 1) using Dipeptide composition method. This table describes the performance of dipeptide composition based SVM model at each threshold (-1 to 1), providing sensitivity, specificity, accuracy and standard error and MCC. Table S5: The performance of SVM model (Learning Parameter: -z c -t 2 -g 0.001 -c 1 -j 1) using Split amino acid composition (2-part) method. This table describes the performance of split amino acid (2-part) composition based SVM model at each threshold (-1 to 1), providing sensitivity, specificity, accuracy and standard error and MCC. Table S6: The performance of SVM model (Learning Parameter: -z c -t 2 -g 0.0001 -c 1 -j 1) using Split amino acid composition (4-part) method. This table describes the perfor [file 1756-0500-4-237-S1.DOC]

**Analysis and prediction of cancerlectins using evolutionary and domain information**

**Additional File-1**

**Table S1:** p-values for compositional differences in cancerlectins and non-cancerlectins residues.

| Amino acids | Cancerlectins Composition | Non-cancerlectins  Composition | p-value |
| --- | --- | --- | --- |
| Alanine (Ala) | 6.855 | 6.418 | 0.15963963 |
| Cysteine (Cys) | 2.758 | 2.686 | 0.72324009 |
| **Aspartic Acid (Asp)** | **4.669** | **5.466** | **0.00290293** |
| Glutamic Acid (Glu) | 6.125 | 5.86 | 0.46065121 |
| Phenylalanine (Phe) | 3.903 | 4.231 | 0.08462309 |
| Glycine (Gly) | 7.423 | 7.647 | 0.47278063 |
| Histidine (His) | 2.313 | 2.151 | 0.33262026 |
| Isoleucine (Ile) | 4.405 | 4.751 | 0.108295 |
| **Lysine (Lys)** | **5.151** | **5.468** | **0.00761836** |
| **Leucine (Leu)** | **9.102** | **8.236** | **0.009655763** |
| Methionine (Met) | 2.071 | 1.873 | 0.15336505 |
| **Asparagine (Asn)** | **4.509** | **5.303** | **0.00367459** |
| **Proline (Pro)** | **5.817** | **4.904** | **0.00758714** |
| Glutamine (Gln) | 4.68 | 4.217 | 0.03682272 |
| **Arginine (Arg)** | **4.865** | **4.209** | **0.00638263** |
| Serine (Ser) | 8.458 | 8.489 | 0.93652765 |
| Threonine (Thr) | 5.981 | 6.355 | 0.19734236 |
| Valine (Val) | 6.043 | 6.347 | 0.05255521 |
| Tryptophan (Trp) | 1.823 | 2.004 | 0.24435425 |
| Tyrosine (Tyr) | 3.05 | 3.382 | 0.04970098 |

**(**Bold values show the residues with significant differences in composition of cancer and non-cancerlectins.**)**

**Table S2:** Performance of BLAST on individual test sets of cancerlectins at E-value cutoff of 0.001.

| **Testsets** | **Total Proteins** | **Total Hits** | **No Hits** | **Correct Hits** | **% coverage** |
| --- | --- | --- | --- | --- | --- |
| 1 | 36 | 28 | 8 | 13 | 36.11 |
| 2 | 36 | 31 | 5 | 13 | 36.11 |
| 3 | 36 | 25 | 11 | 13 | 36.11 |
| 4 | 35 | 27 | 8 | 16 | 45.71 |
| 5 | 35 | 32 | 3 | 19 | 54.29 |

(Note: The five-fold cross validation technique was used for BLAST, in which 4 testsets (consisting of positive and negative proteins) formed the database and the fifth test set was used as a query at E-value cutoff of 0.001.)

**Table S3:** The performance of SVM model (Learning Parameter: -z c –t 2 –g 0.01 –c 0.5 –j 1) using Amino acid composition method.

| **Threshold** | **Sensitivity** | **Specificity** | **Accuracy** | **Std. Error** | **MCC** |
| --- | --- | --- | --- | --- | --- |
| -1.0 | 94.38 | 16.35 | 50.75 | 1.07861207 | 0.16 |
| -0.9 | 91.59 | 25.19 | 54.46 | 1.68705957 | 0.21 |
| -0.8 | 89.35 | 31.83 | 57.18 | 1.1612123 | 0.25 |
| -0.7 | 87.08 | 36.26 | 58.66 | 0.91615283 | 0.27 |
| -0.6 | 83.14 | 43.35 | 60.89 | 0.94253912 | 0.29 |
| -0.5 | 79.21 | 50.89 | 63.36 | 1.07868624 | 0.31 |
| -0.4 | 75.27 | 57.97 | 65.59 | 0.53710334 | 0.33 |
| **-0.3** | **67.97** | **64.15** | **65.84** | **0.93955521** | **0.32** |
| -0.2 | 59.51 | 71.24 | 66.08 | 2.34335571 | 0.31 |
| -0.1 | 52.21 | 74.78 | 64.85 | 1.69668029 | 0.28 |
| 0.0 | 44.87 | 77.88 | 63.36 | 2.12551735 | 0.24 |
| 0.1 | 38.70 | 80.53 | 62.12 | 1.93892083 | 0.21 |
| 0.2 | 33.06 | 82.30 | 60.64 | 1.74465871 | 0.17 |
| 0.3 | 29.14 | 83.18 | 59.40 | 1.85411057 | 0.14 |
| 0.4 | 23.57 | 86.28 | 58.67 | 2.04288864 | 0.13 |
| 0.5 | 17.94 | 88.06 | 57.18 | 1.5617407 | 0.09 |
| 0.6 | 15.14 | 91.59 | 57.93 | 2.02443819 | 0.11 |
| 0.7 | 9.54 | 93.35 | 56.44 | 1.83891925 | 0.06 |
| 0.8 | 6.74 | 94.24 | 55.70 | 1.92436483 | 0.04 |
| 0.9 | 5.62 | 96.45 | 56.44 | 1.58794962 | 0.07 |
| 1.0 | 3.95 | 97.78 | 56.44 | 1.26829413 | 0.06 |

(Bold value indicates the point where overall best result was achieved.)

**Table S4**: The performance of SVM model (Learning Parameter: -z c –t 2 –g 0.001 –c 5 –j 1) using Dipeptide composition method.

| **Threshold** | **Sensitivity** | **Specificity** | **Accuracy** | **Std. Error** | **MCC** |
| --- | --- | --- | --- | --- | --- |
| -1.0 | 94.98 | 12.37 | 48.77 | 1.38234077 | 0.13 |
| -0.9 | 93.86 | 15.91 | 50.25 | 1.06547923 | 0.15 |
| -0.8 | 91.60 | 25.17 | 54.46 | 2.17020183 | 0.21 |
| -0.7 | 87.08 | 32.25 | 56.44 | 1.48087001 | 0.22 |
| -0.6 | 82.56 | 41.13 | 59.40 | 0.99075527 | 0.26 |
| -0.5 | 79.75 | 46.01 | 60.88 | 1.15143649 | 0.27 |
| -0.4 | 75.21 | 53.12 | 62.86 | 1.90852194 | 0.29 |
| **-0.3** | **67.27** | **62.84** | **64.84** | **2.98782128** | **0.30** |
| -0.2 | 58.84 | 69.03 | 64.60 | 2.62439136 | 0.28 |
| -0.1 | 52.67 | 74.33 | 64.85 | 1.74050567 | 0.28 |
| 0.0 | 47.07 | 78.76 | 64.85 | 2.84033625 | 0.28 |
| 0.1 | 37.49 | 81.85 | 62.37 | 4.62120158 | 0.21 |
| 0.2 | 28.00 | 86.28 | 60.65 | 5.13250621 | 0.18 |
| 0.3 | 21.83 | 88.50 | 9.17 | 6.44662284 | 0.14 |
| 0.4 | 16.78 | 89.84 | 57.68 | 7.81502847 | 0.10 |
| 0.5 | 11.21 | 91.17 | 55.95 | 8.83469943 | 0.05 |
| 0.6 | 8.41 | 92.95 | 55.70 | 8.72782527 | 0.03 |
| 0.7 | 6.16 | 94.28 | 55.45 | 9.20294877 | 0.03 |
| 0.8 | 5.59 | 96.47 | 56.44 | 8.82459234 | 0.06 |
| 0.9 | 4.46 | 97.36 | 56.44 | 8.87582526 | 0.04 |
| 1.0 | 3.91 | 97.36 | 56.19 | 8.90266398 | 0.03 |

(Bold value indicates the point where overall best result was achieved.)

**Table S5**: The performance of SVM model (Learning Parameter: -z c –t 2 –g 0.001 –c 1 –j 1) using Split amino acid composition (2-part) method.

(Bold value indicates the point where overall best result was achieved.)

| **Threshold** | **Sensitivity** | **Specificity** | **Accuracy** | **Std. Error** | **MCC** |
| --- | --- | --- | --- | --- | --- |
| -1.0 | 93.27 | 22.57 | 53.72 | 1.33145034 | 0.21 |
| -0.9 | 88.20 | 27.00 | 53.96 | 1.5810914 | 0.19 |
| -0.8 | 87.09 | 32.75 | 56.68 | 1.65861569 | 0.23 |
| -0.7 | 85.40 | 40.26 | 60.14 | 1.09102979 | 0.28 |
| -0.6 | 83.72 | 46.03 | 62.62 | 0.9581837 | 0.32 |
| -0.5 | 77.51 | 53.99 | 64.35 | 0.53852948 | 0.32 |
| -0.4 | 73.05 | 59.32 | 65.35 | 0.71950678 | 0.33 |
| **-0.3** | **66.32** | **64.18** | **65.10** | **0.68139122** | **0.31** |
| -0.2 | 55.60 | 68.59 | 62.87 | 1.00936317 | 0.24 |
| -0.1 | 47.73 | 74.77 | 62.87 | 1.61213213 | 0.23 |
| 0.0 | 39.86 | 77.00 | 60.65 | 2.1069205 | 0.18 |
| 0.1 | 37.64 | 80.10 | 61.40 | 2.31116291 | 0.20 |
| 0.2 | 31.44 | 84.07 | 60.89 | 2.47200647 | 0.18 |
| 0.3 | 26.94 | 87.18 | 60.65 | 2.51265398 | 0.18 |
| 0.4 | 23.59 | 88.50 | 59.91 | 2.38838942 | 0.17 |
| 0.5 | 21.32 | 89.39 | 59.41 | 2.15194935 | 0.16 |
| 0.6 | 18.49 | 91.16 | 59.16 | 1.57553356 | 0.15 |
| 0.7 | 15.16 | 92.49 | 58.43 | 1.36975034 | 0.14 |
| 0.8 | 10.65 | 95.14 | 57.93 | 1.32726787 | 0.12 |
| 0.9 | 7.86 | 96.02 | 57.19 | 1.18798737 | 0.09 |
| 1.0 | 6.17 | 96.91 | 56.94 | 0.81293911 | 0.07 |

**Table S6**: The performance of SVM model (Learning Parameter: -z c –t 2 –g 0.0001 –c 1 –j 1) using Split amino acid composition (4-part) method.

| **Threshold** | **Sensitivity** | **Specificity** | **Accuracy** | **Std. Error** | **MCC** |
| --- | --- | --- | --- | --- | --- |
| -1 | 94.92 | 18.15 | 51.98 | 0.88282728 | 0.2 |
| -0.9 | 91.56 | 25.21 | 54.46 | 1.0410024 | 0.22 |
| -0.8 | 85.92 | 36.27 | 58.16 | 0.780496 | 0.25 |
| -0.7 | 80.89 | 50.46 | 63.86 | 1.39494444 | 0.33 |
| -0.6 | 77.5 | 59.78 | 67.58 | 2.03182529 | 0.38 |
| **-0.5** | **65.12** | **66.85** | **66.09** | **1.86624114** | **0.32** |
| -0.4 | 52.78 | 71.27 | 63.11 | 1.50855693 | 0.25 |
| -0.3 | 44.92 | 77.44 | 63.12 | 1.48927029 | 0.24 |
| -0.2 | 33.13 | 83.63 | 61.39 | 1.37081217 | 0.2 |
| -0.1 | 24.68 | 86.72 | 59.41 | 1.68996154 | 0.16 |
| 0 | 22.46 | 89.83 | 60.16 | 1.12411565 | 0.18 |
| 0.1 | 17.41 | 92.05 | 59.17 | 1.45766114 | 0.16 |
| 0.2 | 12.94 | 95.59 | 59.16 | 1.05396205 | 0.17 |
| 0.3 | 9.01 | 95.59 | 57.43 | 0.78845418 | 0.1 |
| 0.4 | 6.76 | 96.46 | 56.93 | 1.04180324 | 0.08 |
| 0.5 | 3.92 | 97.35 | 56.19 | 0.27544509 | 0.03 |
| 0.6 | 1.11 | 97.8 | 55.2 | 0.38147608 | -0.04 |
| 0.7 | 0 | 98.24 | 54.96 | 0.56082618 | -0.06 |
| 0.8 | 0 | 98.24 | 54.96 | 0.56082618 | -0.06 |
| 0.9 | 0 | 99.12 | 55.45 | 0.312506 | -0.04 |
| 1 | 0 | 99.12 | 55.45 | 0.312506 | -0.04 |

(Bold value indicates the point where overall best result was achieved)

**Table S7**: The performance of SVM model (Learning Parameter: -z c –t 2 –g 7 –c 1 –j 1) using PSSM-based method.

| **Threshold** | **Sensitivity** | **Specificity** | **Accuracy** | **Std Error** | **MCC** |
| --- | --- | --- | --- | --- | --- |
| -1.0 | 92.73 | 17.3 | 50.51 | 2.31580353 | 0.14 |
| -0.9 | 91.06 | 22.61 | 52.76 | 3.83473076 | 0.17 |
| -0.8 | 89.92 | 30.55 | 56.72 | 3.81677351 | 0.24 |
| -0.7 | 87.65 | 35.86 | 58.7 | 3.87443983 | 0.26 |
| -0.6 | 85.41 | 40.29 | 60.19 | 4.35370027 | 0.27 |
| -0.5 | 82.62 | 49.13 | 63.9 | 4.80963575 | 0.33 |
| -0.4 | 77.54 | 56.19 | 65.63 | 3.98193169 | 0.34 |
| -0.3 | 71.89 | 60.14 | 65.38 | 4.08809173 | 0.32 |
| **-0.2** | **67.92** | **68.57** | **68.34** | **2.53125186** | **0.36** |
| -0.1 | 56.68 | 72.99 | 65.86 | 2.31250384 | 0.3 |
| 0.0 | 46.6 | 77.86 | 64.11 | 2.07999663 | 0.26 |
| 0.1 | 42.65 | 80.52 | 63.87 | 1.73345147 | 0.25 |
| 0.2 | 37.03 | 83.19 | 62.87 | 2.60860001 | 0.23 |
| 0.3 | 31.43 | 84.95 | 61.39 | 2.86866066 | 0.2 |
| 0.4 | 26.95 | 86.73 | 60.4 | 2.1362013 | 0.18 |
| 0.5 | 17.99 | 90.73 | 58.67 | 1.46569233 | 0.13 |
| 0.6 | 14.6 | 92.49 | 58.17 | 1.04287775 | 0.12 |
| 0.7 | 12.37 | 94.26 | 58.17 | 1.01415186 | 0.13 |
| 0.8 | 6.17 | 96.02 | 56.44 | 0.54705941 | 0.06 |
| 0.9 | 4.51 | 97.35 | 56.44 | 0.67124064 | 0.06 |
| 1.0 | 3.38 | 98.68 | 56.69 | 0.80362553 | 0.07 |

(Bold value indicates the point where overall best result was achieved)

**Table S8**: The performance of SVM model (Learning Parameter: -z c –t 2 –g 7 –c 5 –j 1) using PSSM-PROSITE domain based method.

| **Threshold** | **Sensitivity** | **Specificity** | **Accuracy** | **Std Error** | **MCC** |
| --- | --- | --- | --- | --- | --- |
| -1 | 93.33 | 15.53 | 49.78 | 3.16984069 | 0.13 |
| -0.9 | 91.63 | 20.41 | 51.76 | 2.86094635 | 0.16 |
| -0.8 | 89.38 | 29.74 | 55.98 | 4.50216326 | 0.22 |
| -0.7 | 86.57 | 36.81 | 58.71 | 4.60636039 | 0.26 |
| -0.6 | 83.75 | 41.23 | 59.94 | 4.70744262 | 0.27 |
| -0.5 | 82.64 | 48.27 | 63.41 | 4.23941812 | 0.32 |
| -0.4 | 80.35 | 54.44 | 65.87 | 2.93361211 | 0.36 |
| -0.3 | 77.54 | 59.75 | 67.6 | 2.66434495 | 0.37 |
| -0.2 | 73.6 | 65.93 | 69.34 | 3.18494176 | 0.39 |
| **-0.1** | **68** | **69.9** | **69.09** | **3.45016724** | **0.38** |
| 0 | 62.97 | 74.32 | 69.34 | 3.48271676 | 0.38 |
| 0.1 | 60.16 | 77.86 | 70.08 | 3.52700581 | 0.39 |
| 0.2 | 55.67 | 78.75 | 68.59 | 3.50745834 | 0.35 |
| 0.3 | 52.3 | 82.31 | 69.09 | 3.68722172 | 0.37 |
| 0.4 | 44.41 | 83.64 | 66.36 | 3.48105587 | 0.31 |
| 0.5 | 37.08 | 87.16 | 65.11 | 1.96940346 | 0.28 |
| 0.6 | 28.11 | 89.82 | 62.63 | 1.7620227 | 0.23 |
| 0.7 | 21.37 | 91.15 | 60.4 | 1.1029116 | 0.18 |
| 0.8 | 17.44 | 93.8 | 60.15 | 1.07899212 | 0.18 |
| 0.9 | 11.25 | 95.57 | 58.42 | 1.25914812 | 0.13 |
| 1 | 9.02 | 96.46 | 57.92 | 1.07996018 | 0.12 |

(Bold value indicates the point where overall best result was achieved.)

**Table S9:** All reported PROSITE domains in cancer and non-cancerlectins.

| No. of PROSITE Domains | Cancerlectins-178 | No. of PROSITE domains | Non-cancerlectin-226 |
| --- | --- | --- | --- |
| 2 | PS01180 | 2 | PS01180 |
| 1 | PS50004 | 1 | PS50022 |
| 1 | PS50017 | 1 | PS50024 |
| 1 | PS50020 | 1 | PS50038 |
| 1 | PS50025 | 1 | PS50082 |
| 1 | PS50027 | 1 | PS50157 |
| 1 | PS50050 | 1 | PS50176 |
| 1 | PS50093 | 1 | PS50215 |
| 1 | PS50095 | 1 | PS50279 |
| 1 | PS50097 | 1 | PS50290 |
| 1 | PS50214 | 1 | PS50294 |
| 1 | PS50215 | 1 | PS50800 |
| 1 | PS50222 | 1 | PS50820 |
| 1 | PS50227 | 1 | PS50847 |
| 1 | PS50228 | 1 | PS50900 |
| 1 | PS50237 | 1 | PS51046 |
| 1 | PS50240 | 1 | PS51073 |
| 1 | PS50261 | 1 | PS51111 |
| 1 | PS50268 | 1 | PS51174 |
| 1 | PS51111 | 1 | PS51189 |
| 1 | PS51132 | 1 | PS51190 |
| 1 | PS51212 | 1 | PS51211 |
| 1 | PS51257 | 1 | PS51233 |
| 2 | PS50024 | 1 | PS51257 |
| 2 | PS50049 | 2 | PS50025 |
| 2 | PS50217 | 2 | PS50027 |
| 2 | PS50221 | 2 | PS50068 |
| 2 | PS50287 | 2 | PS50092 |
| 2 | PS50941 | 2 | PS50234 |
| 2 | PS51092 | 2 | PS50268 |
| 4 | PS50963 | 2 | PS50853 |
| 7 | PS50915 | 2 | PS50941 |
| 7 | PS50923 | 2 | PS50948 |
| 9 | PS50835 | 2 | PS51092 |
| 10 | PS50026 | 2 | PS51115 |
| 24 | PS50231 | 2 | PS51117 |
| 52 | PS50041 | 3 | PS50227 |
|  |  | 3 | PS50240 |
|  |  | 3 | PS50261 |
|  |  | 3 | PS51127 |
|  |  | 3 | PS51132 |
|  |  | 4 | PS50221 |
|  |  | 4 | PS50927 |
|  |  | 7 | PS50228 |
|  |  | 8 | PS50963 |
|  |  | 10 | PS50923 |
|  |  | 12 | PS50835 |
|  |  | 13 | PS50026 |
|  |  | 17 | PS50231 |
|  |  | 61 | PS50041 |
| **Total-151** |  | **Total-200** |  |

All reported domains reported in cancer and non-cancerlectins with their frequency of occurrence. A total of 151 and 200 PROSITE domains were reported in cancer and non-cancer lectins respectively.

**Table S10**: The performance of amino acid composition based SVM model (Learning Parameter: -z c –t 2 –g 0.01 –c 0.5 –j 1) using random dataset of cancer and non-cancerlectins.

| **Threshold** | **Sensitivity** | **Specificity** | **Accuracy** | **MCC** |
| --- | --- | --- | --- | --- |
| -1.0 | 96.55 | 4.03 | 50.63 | 0.04 |
| -0.9 | 95.06 | 7.03 | 51.37 | 0.06 |
| -0.8 | 91.60 | 10.56 | 51.38 | 0.05 |
| -0.7 | 87.15 | 13.06 | 50.39 | 0.02 |
| -0.6 | 85.15 | 17.08 | 51.39 | 0.04 |
| -0.5 | 81.68 | 21.60 | 51.89 | 0.04 |
| -0.4 | 76.23 | 29.18 | 52.88 | 0.06 |
| -0.3 | 76.23 | 29.18 | 52.88 | 0.06 |
| -0.2 | 68.30 | 41.21 | 54.88 | 0.10 |
| -0.1 | 61.40 | 46.24 | 53.89 | 0.08 |
| **0.0** | **57.93** | **50.77** | **54.38** | **0.09** |
| 0.1 | 49.46 | 56.82 | 53.12 | 0.07 |
| 0.2 | 44.99 | 61.35 | 53.12 | 0.06 |
| 0.3 | 40.04 | 68.37 | 54.11 | 0.09 |
| 0.4 | 34.61 | 73.92 | 54.12 | 0.10 |
| 0.5 | 23.71 | 76.42 | 49.87 | 0.00 |
| 0.6 | 17.76 | 83.45 | 50.36 | 0.01 |
| 0.7 | 11.83 | 87.47 | 49.37 | -0.01 |
| 0.8 | 7.88 | 90.50 | 48.88 | -0.02 |
| 0.9 | 6.90 | 93.00 | 49.62 | 0.00 |
| 1.0 | 3.95 | 96.50 | 49.87 | 0.01 |

(Bold value indicates the point where overall best result was achieved.)
